# Influence of beta-blocker therapy on the risk of infections and death in patients at high risk for stroke induced immunodepression

**DOI:** 10.1371/journal.pone.0196174

**Published:** 2018-04-25

**Authors:** Ilko L. Maier, Johannes C. Becker, Johanna Rosemarie Leyhe, Marlena Schnieder, Daniel Behme, Marios-Nikos Psychogios, Jan Liman

**Affiliations:** 1 Department of Neurology, University Medical Center Göttingen, Göttingen, Germany; 2 Department of Neurology, St. Bernward Hospital Hildesheim, Hildesheim, Germany; 3 Department of Neuroradiology, University Medical Center Göttingen, Göttingen, Germany; Osaka University Graduate School of Medicine, JAPAN

## Abstract

**Background:**

Stroke-induced immunodepression is a well characterized complication of acute ischemic stroke. In experimental studies beta-blocker therapy reversed stroke-induced immunodepression, reduced infection rates and mortality. Recent, heterogeneous studies in stroke patients could not provide evidence of a protective effect of beta-blocker therapy. Aim of this study is to investigate the potential preventive effect of beta-blockers in subgroups of patients at high risk for stroke-induced immunodepression.

**Methods:**

Data from a prospectively derived registry of major stroke patients receiving endovascular therapy between 2011–2017 in a tertiary stroke center (University Medical Center Göttingen. Germany) was used. The effect of beta-blocker therapy on pneumonia, urinary tract infection, sepsis and mortality was assessed using multivariate logistic regression analysis.

**Results:**

Three hundred six patients with a mean age of 72 ± 13 years and a median NIHSS of 16 (IQR 10.75–20) were included. 158 patients (51.6%) had pre-stroke- and continued beta-blocker therapy. Beta-blocker therapy did not reduce the incidence of pneumonia (OR 0.78, 95% CI 0.31–1.92, p = 0.584), urinary tract infections (OR 1.51, 0.88–2.60, p = 0.135), sepsis (OR 0.57, 0.18–1.80, p = 0.334) or mortality (OR 0.59, 0.16–2.17, p = 0.429). Strokes involving the insula and anterio-medial cortex increased the risk for pneumonia (OR 4.55, 2.41–8.56, p<0.001) and sepsis (OR 4.13, 1.81–9.43, p = 0.001), while right hemispheric strokes increased the risk for pneumonia (OR 1.60, 0.92–2.77, p = 0.096). There was a non-significantly increased risk for urinary tract infections in patients with beta-blocker therapy and insula/anterio-medial cortex strokes (OR 3.12, 95% CI 0.88–11.05, p = 0.077) with no effect of beta-blocker therapy on pneumonia, sepsis or mortality in both subgroups.

**Conclusions:**

In major ischemic stroke patients, beta-blocker therapy did not lower post-stroke infection rates and was associated with urinary tract infections in a subgroup with insula/anterio-medial strokes.

## Introduction

Infections are frequent and decisive complications after a stroke, as they have a significant influence on stroke outcome [1–3]. Up to a third of all stroke patients subsequently develop infectious complications with pneumonia and urinary tract infections accounting for the majority of cases [4]. As a result of stroke, these patients often show risk factors such as immobilization and dysphagia, which may promote the development of such an infection [5, 6]. However, it has been recognized that stroke patients develop considerably higher rates of infections compared to orthopedic patients with comparable degrees of functional impairments and comorbidities [7]. In this respect, studies have established a link between a damage to the central nervous system and peripheral immunomodulation. Firstly, stroke-associated tissue damage can affect the interaction between the innate and adaptive immune system and hereby impair the specific immune response to bacterial pathogens [8–10]. Secondly, stroke causes the release of stress hormones such as glucocorticoids and catecholamines. These have an immunodepressive effect and influence the functionality of immune cells [9, 11]. The latter finding lead to experimental studies on the effect of beta-antagonists in stroke models.

In an experimental study, Prass et al. found that the administration of propranolol to middle cerebral artery occlusion (MCAO) mice reinforced the immune response and lowered infection rates [11]. Other experimental studies indicated that hepatic invariant natural killer T (INKT)- cells could play an important role in the immunomodulating pathway. In MCAO mice, INKT cells showed an anti-inflammatory phenotype causing higher rates of infections and mortality. The authors were able to reverse this phenotype by the administration of propranolol, which significantly reduced rates of infections and mortality [12].

Previous studies involving humans, however, were inconclusive. Some described a protective effect of beta-blockers regarding infection rates [13–15], others showed a negative effect of beta-blockers with higher rates of adverse events [16].

The main idea behind previous experimental and clinical studies was to antagonize the immunodepressive effects caused by an increase in the sympathetic tone with beta receptor antagonists. This theory has not been sufficiently studied in stroke patients at high risk for infections and in patients likely to exhibit the immunodepressive mechanism previously described in several studies. Our aim was to study the possible immunomodulatory effect of beta-blockers in different subgroups of patients with major ischemic strokes.

## Materials and methods

### Patient population and clinical characteristics

Clinical and neuroradiological data were analyzed from a prospectively derived, monocentric database including neuroradiological and neurological information of patients with endovascular treatment. The database also included detailed information on pre-stroke medication, past medical history and post-stroke complications. Infection related risk factors like the development of dysphagia, naso-gastric tubes and transurethral catheters were obtained retrospectively through a chart review (IntelliSpace Critical Care and Anesthesia (ICCA) information system (Koninklijke Philips N.V., 2004–2017)), in which detailed information on the clinical course of the stroke patients were documented by stroke physicians and therapists. Ethics approval for this study was sought from the Ethics Committee of the University Medicine Göttingen and all patients or next of kin gave informed written consent for the anonymized use of disease-related data on hospitalization.

All patients were admitted to a neurointensive care unit directly after endovascular treatment and transferred to a stroke unit at various timepoints depending on their clinical status. Patients receiving beta-blockers before the stroke and continued beta-blocker therapy during their in-hospital stay were considered as beta-blocker positive. Patients without beta-blocker exposure before the stroke or during in-patient stay were considered as beta-blocker negative. Patients were included in the analysis, if all necessary data on pre-stroke history (comorbidities and pre-stroke medication)- and clinical data on post stroke course (e.g. infections or complications) were complete and available, which was the case in 306 (59%) from 518 documented cases in the prospectively derived databank.

We defined two high risk subgroups of stroke patients at increased risks for stroke induced immunodepression and post-stroke infections. The first group had insula and anterio-medial cortex infarctions, which have been identified to have an increased sympathetic tone and higher risk for post-stroke infections in other studies [17,18]. We assessed the localization of the stroke by using the Alberta Stroke Program Early CT-Score (ASPECTS) [19]. The M1- and M4-regions were defined as antero-medial cortex. The second group with a higher risk for infections were right hemispheric strokes, which in previous studies have been shown to have an increased sympathetic tone [20,21].

### Post-stroke infections

Following infectious complications were investigated: pneumonia, urinary tract infection and sepsis. For the diagnosis of these infections, the recommendations of the current German guidelines were followed. For the diagnosis of pneumonia, these included a newly diagnosed infiltrate/suspicion of an infiltrate on chest X-ray in presence of at least two clinical signs such as cough, fever (>38.0°C), leukocytosis or leukopaenia (>10,000 or <4,000/l) or the detection of a pneumonia-typical pathogen in the sputum, bronchial secretion or blood [22].

Criteria for the diagnosis of an urinary tract infection included a nitrite-positive urine test strip, bacteria in the midstream urine (10^3^ colony-forming unit (CFU)/ml with symptoms or 10^5^ CFU/ ml without symptoms) or a positive urine culture with bacteria typical of urinary tract infections [23]. Sepsis was diagnosed, if the sequential (sepsis-related) organ failure assessment (SOFA) score was ≥ 2 in combination with positive blood cultures [24].

### Statistical analysis

Statistical analysis was performed using SPSS 21 (IBM SPSS Statistics, Armonk, NY, USA). Characteristics of all patients are shown as mean ± standard deviation (SD), if normally distributed, and as median with interquartile range (IQR), if not (Shapiro-Wilk test for normal distribution). Category variables were given as absolute frequencies and percentages and examined by Pearson Chi-Square test for statistically significant differences between the compared groups. The different groups were examined for significant differences by using independent samples T-test or Mann–Whitney U test, as appropriate. Furthermore, to assess the influence of beta-blocker therapy on post-stroke infections, an uni- and multivariate logistic regression was carried out. Relevant confounders (being predictive for the respective infectious endpoint with a p-value <0.2 in an univariate pre-test) were included in a multivariate logistic regression together with the predictor beta-blocker therapy. This logistic regression was performed as an inclusion procedure. In all procedures, a p-value of <0.05 was considered as statistically significant.

## Results

### Baseline characteristics

Patients in the beta-blocker positive group were older (p<0.001), were more likely to be female (p<0.001), to have a history of arterial hypertension (p<0.001), coronary heart disease (<0.001), atrial fibrillation (<0.001) and chronic renal failure (p = 0.006, Table 1). There was no significant difference of National Institute of Health Stroke Scale (NIHSS) on admission (median NIHSS 17 vs 15, p = 0.068), but patients with beta-blocker therapy had a higher modified Rankin Scale (mRS) on admission (median mRS 5 vs 4, p = 0.007) and a higher NIHSS 48 hours after stroke (17 vs 13.5, p = 0.014) compared to patients without beta-blocker therapy. Patients in the beta-blocker positive group were more likely to develop dysphagia after the stroke (80,9% vs 66,9%, p = 0.006). Rates of decompressive hemicraniectomy and any other interventions (like surgeries or invasive diagnostic procedures) were more frequent in the beta-blocker negative group. Eighty-three patients (52,5%) received bisoprolol, 54 patients (38%) metoprolol, 4 patients (5,1%) nebivolol, 3 patients (2,5%) carvedilol, 2 patients (1,9%) atenolol.

**Table 1:** Baseline characteristics and distribution of potential confounding factors of patients with- and without beta-blocker therapy.

	Beta-Blocker	No-Beta-Blocker	p-value
	(n = 158)	(n = 148)	
Age (mean years ± SD)	77 (71–83)	69 (58–78)	<0.001
Sex (male, %)	58 (36.7)	88 (59.5)	<0.001
Arterial hypertension (n, %)	153 (96.8)	91 (61.5)	<0.001
Hyperlipidemia (n, %)	73 (48)	61 (41.8)	0.296
Diabetes mellitus (n, %)	53 (34)	38 (25.9)	0.134
Atrial fibrillation (n, %)	89 (57.4)	36 (24.7)	<0.001
Peripheral arterial disease (n, %)	13 (8.4)	6 (4.1)	0.157
Coronary heart disease (n, %)	57 (36.5)	17 (11.6)	<0.001
Chronic renal failure (n, %)	35 (25)	17 (12.1)	0.006
NIHSS admission (median points, IQR)	17 (11–21)	15 (10–19)	0.068
NIHSS 48 hours post admission (median points, IQR)	17 (9–24)	13.5 (5–22)	0.014
mRS admission (median points, IQR)	5 (4–5)	4 (4–5)	0.007
Successful recanalization (n, %)	117 (74.5)	105 (70.9)	0.521
In-hospital days (median, IQR)	9 (6–18.25)	10 (6–19)	0.849
Immunodepressive medication (n, %)	8 (5.1)	17 (11.5)	0.059
Naso-gastric tube (n, %)	84 (53.2)	72 (49)	0.493
Dysphagia (n, %)	127 (80.9)	99 (66.9)	0.006
Transurethral catheter (n, %)	149 (94.3)	131 (88.5)	0.099
Intubation (n, %)	93 (58.9)	100 (67.6)	0.124
Length of intubation (median days, IQR)	1 (1–2)	1 (1–3)	0.157
Tracheotomy (n, %)	11 (7)	15 (10.1)	0.413
Time from stroke to tracheotomy (median days, IQR)	10 (8–19)	14 (11–17)	0.443
Other intervention (n, %)	21 (13.3)	34 (23)	0.036
Hemicraniectomy (n, %)	7 (4.4)	18 (12.1)	0.022

SD: standard deviation; IQR: interquartile range; Other interventions: non-stroke related major surgical- or invasive diagnostic procedures; Immunodepressive medication: premedication with any drugs that inhibit or prevent activity of the immune system

### Effect of beta-blocker therapy on infection rates

Considering the total patient population, 84 (27.4%) developed pneumonia, 80 (26.1%) an urinary tract infection and 40 (13.1%) sepsis. 64 (20.9%) of the patients died during their in-patient stay.

As shown in Table 2, there was no difference in rates of pneumonia, sepsis and mortality between the beta blocker positive and negative group. Urinary tract infections were more frequently diagnosed in the beta-blocker positive group (50 (31.6%) vs 30 (20.3%) patients, p = 0.027).

**Table 2:** Infection rates and mortality in patients with- and without beta-blocker therapy.

	Beta-Blocker (n = 158)	No-Beta-Blocker (n = 148)	Overall (n = 306)	p-value
Pneumonia (n, %)	44 (27.8%)	40 (27%)	84 (27.4%)	0.898
Urinary tract infection (n, %)	50 (31.6%)	30 (20.3%)	80 (26.1%)	0.027
Sepsis (n, %)	22 (13.9%)	18 (12.2%)	40 (13.1%)	0.735
Mortality (n, %)	38 (24.1%)	26 (17.6%)	64 (20.9%)	0.206

Table 3 shows the univariate and multivariate logistic regression of beta-blocker therapy and confounding factors on post-stroke infections and mortality. The univariate analysis showed no effect of beta blocker therapy on pneumonia (OR 1.04, 95% CI 0.63–1.72, p = 0.874), sepsis (OR 1.17, 95% CI 0.65–2.28, p = 0.648) or mortality (OR 1.47, 95% CI 0.84–2.58, p = 0.178). This was also the case for the multivariate logistic regression, which included relevant confounding factors for these infectious endpoints like dysphagia, length of intubation or NIHSS.

**Table 3:** Uni- and multivariate logistic regression models for the association between beta-blocker therapy, post-stroke infections and mortality.

	Univariable analysis		Multivariable analysis	
	OR (95% CI)	p-value	OR (95% CI)	p-value
*Pneumonia*				
Beta-blocker	1.04 (0.63–1.72)	0.874	0.78 (0.31–1.92)	0.584
Naso-gastric tube			2.00 (0.76–5.30)	0.162
NIHSS 48 hours post admission			0.95 (0.89–1.02)	0.144
Intubation			0.64 (0.23–1.77)	0.388
Length of intubation			0.82 (0.66–1.02)	0.070
Tracheotomy			0.34 (0.02–7.47)	0.490
Other intervention			3.84 (1.02–14.45)	0.046
Dysphagia			5.46 (0.54–55.35)	0.151
Age			0.99 (0.94–1.03)	0.570
Diabetes mellitus			2.56 (1.06–6.20)	0.037
Hemicraniectomy			0.59 (0.05–6.99)	0.679
Successful recanalization			0.88 (0.33–2.38)	0.814
mRS admission			1.16 (0.70–1.93)	0.570
Insula and antero-medial cortex			2.45 (0.93–6.45)	0.069
Right hemispheric stroke			1.64 (0.68–3.99)	0.273
*Urinary tract infections*				
Beta-blocker	1.82 (1.08–3.07)	0.025	1.51 (0.88–2.60)	0.135
Sex			0.54 (0.32–0.94)	0.028
NIHSS admission			0.98 (0.94–1.02)	0.259
Transurethral catheter			1.75 (0.57–5.37)	0.328
*Sepsis*				
Beta-blocker	1.17 (0.65–2.28)	0.648	0.57 (0.18–1.80)	0.334
Naso-gastric tube			1.77 (0.52–5.99)	0.362
NIHSS 48 hours post admission			0.97 (0.89–1.05)	0.388
Intubation			2.71 (0.51–14.47)	0.243
Length of intubation			0.79 (0.65–0.98)	0.031
Tracheotomy			6.71 (0.43–105.41)	0.176
Diabetes mellitus			1.45 (0.44–4.74)	0.542
Hemicraniectomy			0.07 (0.004–1.37)	0.080
Successful recanalization			0.88 (0.24–3.24	0.850
mRS admission			1.10 (0.61–1.99)	0.746
Insula and antero-medial Cortex			3.48 (0.93–13.03)	0.064
*Mortality*				
Beta-blocker	1.47 (0.84–2.58)	0.178	0.59 (0.16–2.17)	0.429
Sepsis			3.85 (0.76–19.61)	0.104
Immunosuppressive medication			1.96 (0.24–16.139	0.531
Naso-gastric tube			0.27 (0.07–1.00)	0.050
Transurethral catheter			0.02 (0.00–1.56)	0.080
Intubation			0.78 (0.21–2.87)	0.702
Length of intubation			0.84 (0.68–1.04)	0.109
Tracheotomy			0.02 (0.00–0.97)	0.048
Dysphagia			12.14 (0.24–611.25)	0.212
Age			0.87 (0.80–0.94)	0.001
Diabetes mellitus			1.14 (0.30–4.319	0.850
Chronic kidney failure			1.55 (0.41–5.85)	0.522
NIHSS admission			1.06 (0.94–1.21)	0.347
Successful recanalization			0.35 (0.10–1.31)	0.119
mRS admission			0.98 (0.39–2.47)	0.968
Right hemispheric stroke			0.57 (0.18–1.80)	0.341
Insula and antero-medial cortex			16.75 (4.25–65.94)	<0.001

OR: Odds Ratio; CI: confidence interval; Other interventions: non-stroke related major surgical- or inva-sive diagnostic procedures; Immunodepressive medication: premedication with any drugs that inhibit or prevent activity of the immune system

Beta blocker therapy was associated with a significantly increased risk for urinary tract infection in the univariate (OR 1.82, 95% CI 1.08–3.07, p = 0.025), but not in the multivariate logistic regression (OR 1.51, 95% CI 0.88–2.60, p = 0.135) analysis.

Patients with- and without beta-blocker therapy showed no difference in leukocyte count, C-reactive protein or procalcitonin concentration (S1 Table).

### Influence of stroke localization

Patients with strokes affecting the insula and antero-medial cortex showed higher rates of pneumonia (OR 4.55, 95% CI 2.41–8.56, p<0.001), sepsis (OR 4.13, 95% CI 1.81–9.43, p = 0.001) and mortality (OR 13.11, 95% CI 5.41–31.77, p<0.001) compared to patients with other stroke localizations. This stroke localization did not influence the risk for urinary tract infections (OR 1.09, 95% CI 0.59–1.99, p = 0.788).

As shown in Table 4, there was a higher number of patients with an urinary tract infection in the beta-blocker group compared to the non-beta-blocker group (18 (40%) vs 6 (18.8%), p = 0.079) resulting in an increased risk for urinary tract infections in the beta-blocker group (adjusted OR 3.12, 95% CI 0.88–11.05, p = 0.077), which did not reach statistical significance. There was no difference between the beta-blocker positive and beta-blocker negative group for pneumonia, sepsis or mortality in this subgroup.

**Table 4:** Pneumonia, urinary tract infection, sepsis and mortality in patients with insula/anterio-medial strokes with- and without beta-blocker therapy.

	Beta-Blocker	No-Beta-Blocker	p-value
*Insula/anterio-medial strokes (n = 77)*			
Pneumonia (n, %)	19 (42.2)	18 (56.3)	0.254
Urinary tract infections (n, %)	18 (40)	6 (18.8)	0.079
Sepsis (n, %)	11 (24.4)	8 (25)	1.000
Mortality (n, %)	18 (40)	14 (43.8)	0.816
*Right hemispheric strokes(n = 113)*			
Pneumonia (n, %)	17 (32.7)	19 (31.1)	1.000
Urinary tract infections (n, %)	17 (32.7)	12 (19.7)	0.134
Sepsis (n, %)	5 (9.6)	9 (14.8)	0.569
Mortality (n, %)	10 (19.2)	6 (10)	0.186

### Influence of the side of stroke

There was a non-significant increased risk for pneumonia in patients with right hemispheric strokes (OR 1.60, 95% CI 0.92–2.77, p = 0.096), while there was no influence of right sided strokes on urinary tract infection (OR 0.99, 95% CI 0.57–1.73, p = 0.976) or sepsis (OR 0.98, 95% CI 0.47–2.06, p = 0.963). Right hemispheric strokes were associated with lower mortality (OR 0.57, 95% CI 0.28–1.02, p = 0.057).

In the subgroup of patients with right sided strokes, there was no difference for pneumonia, urinary tract infections, sepsis or mortality between patients with- and without beta-blocker therapy (Table 4).

## Discussion

In our study, we found no effect of beta-blocker therapy on rates of post-stroke pneumonia, sepsis or mortality in patients at high risk for stroke induced immunodepression and post-stroke infectious complications, with even increased rates for urinary tract infections. These findings were also true for anatomically defined subgroups with insular/ antero-medial cortex and right hemispheric strokes, which have been shown to have increased risks for post-stroke infections and stroke induced immunodepression [17,18].

One general reason for the incapability of beta-blockers to prevent post-stroke immunodepression may be the fact that nowadays mainly cardioselective (beta-1 receptor selective) beta-blockers are used. Previous studies provided evidence, that immune cells may be modulated primarily via beta 2 receptors (rather via beta-1-receptors) by catecholamines [25]. This could be the reason why we could not find protective effects of pre-stroke beta blocker therapy, also given the fact that previous experimental studies demonstrated a protective effect of unselective beta-blockers only [11,12]. Two non-experimental studies found a protective effect of beta-blockers therapy for pneumonia-rates. Dziedzic et al. discussed their findings with a higher rate of unselective beta-blocker use [13] and Sykora et al. was able to reproduce these findings with a comparable distribution of beta-blocker types [14]. These studies indicate, that unselective beta-blockers, as shown in multiple experimental studies, might play a role in inhibiting stroke induced immunodepressive mechanisms, while beta-1-selective beta blockers don’t. On the contrary, however, these findings were not confirmed by two recent studies using prospectively derived data from patients with ischemic- and hemorrhagic stroke receiving unselective beta-blockers [26, 27]. In conclusion, the role of beta-2-antagonism to prevent post-stroke infections remains unclear.

A recent subgroup analysis of the Preventive Antibiotics in Stroke Study (PASS)-study also could not provide evidence for a protective effect of beta blocker therapy on infections, showing even increased pneumonia-rates [16]. However, the diagnosis of infections in this study was based on the judgement of the treating physician, the authors did not differentiate between pre- and continued beta-blocker therapy, did not distinguish between selective- and non-selective beta-blockers and the NIHSS at baseline was lower compared to our study (5 vs 16 points). Given these differences, it can be argued that, on the one hand, the pneumonia rates might have been overestimated and, on the other, the number of patients with stroke-induced immunodepression were lower (due to lower stroke volumes). However, our findings basically reproduce the findings of the PASS-subanalysis and therefore indicate that there are no differential effects of beta-blocker therapy in subgroups of minor and major stroke patients.

The increased risk for urinary tract infections associated with beta-blocker therapy could be explained by the fact, that only major stroke patients were studied, and the diagnosis of urinary tract infections was primarily based on bacteriological and urinary status in combination with signs of infections rather than clinical signs attributable to urinary tract infection. These can’t be judged in the majority of cases in major stroke patients, showing aphasia, confusion and decreased conscious state. Therefore, rates of urinary tract infection might be overestimated in our study. The exact mechanism, by which beta-blockers might contribute to an increased rate of urinary tract infections, remains unclear. Given the pleiotropic and complex effects of beta-blockade on the immune system on the one-, and sympathetic nervous system and multitude of organ systems on the other hand, protective effects of beta-blocker therapy might be outweighed by detrimental effects of beta-blocker therapy. However, considering the problem of proper diagnosis and possibility of overestimation of urinary tract infection rates, our study (together with previous studies reporting increased infection rates) provides safety concerns for the use of beta-blockers in patient with ischemic stroke.

The strength of the study lies in the fact that we included a homogeneous patient group at high risk for post-stroke infections and stroke-induced immunodepression, which has not been studied before. Moreover, we were able to correct for major confounding factors like naso-gastric tubes, intubation and dysphagia, therefore providing more robust results compared to previous studies [15]. Major limitations of our study include the monocentric design as well as the lack of included patients with unselective beta-blockers. As detailed pre- and post-stroke data were incomplete in a significant proportion of patients in the database, selection bias can’t be ruled out. Lack of statistical power might play a role in the multivariate logistic regression, as multiple confounders have been included. Moreover, the problem of survival bias should be noted, as very severely affected patients that do not survive the hyperacute phase after stroke or do receive palliative treatment do not develop post-stroke infections, which they might have developed if they did survive the hyperacute phase. Another limitation is that general diagnosis criteria for hospital-acquired pneumonias have been used in this study and not criteria, which specifically have been proposed for the diagnosis of stroke-associated pneumonia by the Pneumonia in Stroke Consensus Group [28].

In conclusion, we could not provide evidence for a protective effect of beta-blockers on post stroke infections and mortality. Data in humans remain inconclusive with studies showing protective effects on pneumonia rates [13, 14] and mortality [29] while other studies show neutral effects [30] or increased rates of infections [16]. The latter may raise safety concerns of these commonly prescribed group of antihypertensive drugs. Our study, however, provides evidence for the safety of beta-blocker therapy concerning the most severe post stroke complications pneumonia, sepsis and death in a major stroke patient group. The increased rate of urinary tract infections in patients with insula/anterio-medial strokes associated with beta-blocker therapy, however, should be considered when choosing antihypertensive drugs in major stroke patients at high risk for such infections.

Further studies should investigate the possible effect of unselective beta-blockers on post stroke infections using a prospective design to optimize the control for confounders and should not only identify high risk patients for stroke induced immunodepression using radiological data, but also include serological measurements of catecholamines and cortisol as well as continuous measurements of heart rate and blood pressure to identify an increased sympathetic tone.

## Supporting information

S1 Table Leukocyte count, C-reactive protein and procalcitonin in patients with- and without beta-blocker therapy. (DOCX)
