# Supplementary material for: Influence of beta-blocker therapy on the risk of infections and death in patients at high risk for stroke induced immunodepression
Source: PLoS One. 2018 Apr 25;13(4):e0196174. doi: 10.1371/journal.pone.0196174 (PMC5919008; doi:10.1371/journal.pone.0196174)
Supplement: S1 Table — (DOCX) [file pone.0196174.s001.docx]

**Data Supplement**

| S1 Table: Leukocyte count, C-reactive protein and procalcitonin in patients with- and without beta-blocker therapy | | | |
| --- | --- | --- | --- |
|  |  |  |  |
|  | Beta-blocker positive | Beta-blocker negative | p-value |
| Maximum leukocyte count (*10^3 ± SD) | 13.5 ± 5.2 | 13.5 ± 5.7 | 0.936 |
| Delta leukocyte count (*10^3 ± SD) | 6.3 ± 4.6 | 6.2 ± 4.9 | 0.861 |
| Maximum C-reactive protein (mg/dl ± SD) | 53.2 ± 61.7 | 55.7 ± 72.5 | 0.766 |
| Delta C-reactive protein (mg/dl ± SD) | 38.7 ± 56.1 | 41.9 ± 69.7 | 0.667 |
| Maximum Procalcitonin (µg/l ± SD; n=219) | 1.2 ± 3.5 | 1.1 ± 3.7 | 0.875 |
| Delta Procalcitonin (µg/l ± SD; n=219) | 1 ± 3.4 | 0.9 ± 3.7 | 0.920 |
| SD: Standard deviation; Delta: maximum minus minimum parameter | | |  |
|  |  |  |  |

| Individual patient data (n=306) | | | | | | |
| --- | --- | --- | --- | --- | --- | --- |
|  |  |  |  |  |  |  |
| Age (years) | NIHSS (baseline) | NIHSS (48 h follow up) | mRS (baseline) | length of intubation (days) | In-hospital days | Days from stroke to tracheotomy |
| 60 | 4 | 0 | 5 | 1 | 7 |  |
| 58 | 19 | 20 | 4 | 0 | 3 |  |
| 81 | 9 | 3 | 5 | 0 | 4 |  |
| 69 | 12 | 6 | 4 | 0 | 2 |  |
| 64 | 14 | 13 | 4 | 15 | 25 | 18 |
| 77 | 18 | 8 | 4 | 0 | 8 |  |
| 46 | 18 | 23 | 5 | 13 | 21 | 14 |
| 77 | 24 | 23 | 5 | 13 | 17 | 15 |
| 76 | 19 | 23 | 4 | 0 | 2 |  |
| 88 | 14 | 17 | 4 | 0 | 4 |  |
| 81 | 13 | 5 | 3 | 0 | 4 |  |
| 56 | 7 | 0 | 5 | 0 | 4 |  |
| 60 | 20 | 5 | 4 | 0 | 4 |  |
| 76 | 16 | 7 | 3 | 1 | 5 |  |
| 78 | 7 | 2 | 2 | 0 | 8 |  |
| 66 | 5 | 1 | 4 | 0 | 7 |  |
| 71 | 17 | 5 | 2 | 0 | 4 |  |
| 83 | 4 | 3 | 4 | 1 | 19 |  |
| 49 | 10 | 2 | 5 | 0 | 4 |  |
| 56 | 17 | 16 | 4 | 6 | 22 |  |
| 74 | 15 | 13 | 2 | 0 | 13 |  |
| 82 | 3 | 25 | 4 | 6 | 7 |  |
| 56 | 11 | 7 | 5 | 1 | 8 |  |
| 89 | 20 | 22 | 5 | 0 | 4 |  |
| 73 | 24 | 19 | 5 | 2 | 3 |  |
| 74 | 17 | 23 | 4 | 3 | 2 |  |
| 60 | 17 | 8 | 5 | 0 | 7 |  |
| 45 | 26 | 26 | 5 | 13 | 14 | 13 |
| 75 | 18 | 17 | 4 | 0 | 7 |  |
| 82 | 12 | 4 | 5 | 0 | 7 |  |
| 78 | 19 | 11 | 4 | 0 | 4 |  |
| 50 | 12 | 4 | 5 | 1 | 5 |  |
| 62 | 11 | 16 | 5 | 1 | 20 |  |
| 53 | 32 | 32 | 1 | 7 | 7 |  |
| 63 | 5 | 23 | 5 | 18 | 24 | 22 |
| 85 | 23 | 1 | 4 | 0 | 5 |  |
| 69 | 11 | 27 | 5 | 2 | 2 |  |
| 74 | 22 | 19 | 5 | 17 | 9 | 17 |
| 73 | 12 | 5 | 5 | 0 | 13 |  |
| 57 | 12 | 9 | 4 | 1 | 35 |  |
| 89 | 15 | 8 | 4 | 1 | 13 |  |
| 78 | 9 | 3 | 3 | 0 | 7 |  |
| 56 | 8 | 10 | 5 | 0 | 16 |  |
| 57 | 16 | 0 | 4 | 0 | 4 |  |
| 70 | 13 | 4 | 3 | 0 | 4 |  |
| 81 | 7 | 1 | 4 | 1 | 7 |  |
| 83 | 7 | 15 | 5 | 1 | 9 |  |
| 84 | 35 | 16 | 5 | 1 | 26 |  |
| 89 | 18 | 21 | 5 | 1 | 16 |  |
| 77 | 23 | 20 | 2 | 1 | 10 |  |
| 28 | 10 | 3 | 5 | 0 | 8 |  |
| 48 | 27 | 14 | 5 | 11 | 22 | 11 |
| 58 | 15 | 15 | 3 | 1 | 21 |  |
| 46 | 3 | 0 | 5 | 0 | 7 |  |
| 61 | 32 | 27 | 5 | 17 | 18 |  |
| 76 | 17 | 32 | 5 | 4 | 3 |  |
| 68 | 14 | 9 | 5 | 0 | 6 |  |
| 52 | 19 | 16 | 5 | 1 | 12 |  |
| 62 | 18 | 15 | 4 | 4 | 30 |  |
| 52 | 11 | 22 | 5 | 1 | 1 |  |
| 78 | 18 | 16 | 5 | 1 | 24 |  |
| 80 | 22 | 28 | 4 | 2 | 3 |  |
| 80 | 10 | 3 | 4 | 1 | 4 |  |
| 53 | 11 | 4 | 5 | 0 | 13 |  |
| 59 | 23 | 20 | 4 | 0 | 9 |  |
| 76 | 13 | 12 | 4 | 1 | 23 |  |
| 87 | 15 | 2 | 3 | 1 | 7 |  |
| 71 | 5 | 1 | 4 | 0 | 10 |  |
| 76 | 15 | 10 | 5 | 0 | 8 |  |
| 69 | 22 | 15 | 5 | 1 | 26 |  |
| 65 | 22 | 5 | 4 | 1 | 16 |  |
| 85 | 12 | 5 | 5 | 1 | 10 |  |
| 76 | 20 | 21 | 5 | 13 | 7 | 20 |
| 63 | 10 | 30 | 5 | 1 | 21 |  |
| 77 | 12 | 14 | 4 | 8 | 20 |  |
| 68 | 8 | 8 | 5 | 12 | 54 | 12 |
| 59 | 9 | 14 | 2 | 1 | 19 |  |
| 44 | 7 | 5 | 4 | 0 | 9 |  |
| 84 | 10 | 22 | 0 | 7 | 17 |  |
| 60 | 5 | 32 | 5 | 6 | 31 |  |
| 77 | 23 | 2 | 5 | 1 | 8 |  |
| 79 | 12 | 13 | 4 | 3 | 13 |  |
| 30 | 16 | 0 | 5 | 1 | 9 |  |
| 78 | 18 | 18 | 3 | 3 | 2 |  |
| 76 | 20 | 14 | 4 | 2 | 21 |  |
| 78 | 19 | 2 | 5 | 1 | 11 |  |
| 52 | 12 | 9 | 4 | 0 | 28 |  |
| 80 | 15 | 15 | 3 | 2 | 18 |  |
| 42 | 9 | 0 | 5 | 0 | 18 |  |
| 72 | 18 | 4 | 5 | 1 | 9 |  |
| 60 | 9 | 21 | 2 | 6 | 8 |  |
| 62 | 7 | 4 | 5 | 0 | 15 |  |
| 56 | 18 | 10 | 4 | 1 | 13 |  |
| 58 | 16 | 4 | 5 | 0 | 4 |  |
| 46 | 12 | 10 | 4 | 1 | 10 |  |
| 67 | 16 | 6 | 4 | 0 | 4 |  |
| 65 | 28 | 22 | 5 | 6 | 4 |  |
| 80 | 18 | 28 | 4 | 1 | 2 |  |
| 53 | 18 | 21 | 4 | 6 | 29 | 6 |
| 81 | 20 | 31 | 3 | 1 | 16 |  |
| 45 | 11 | 9 | 4 | 1 | 6 |  |
| 77 | 21 | 17 | 5 | 1 | 8 |  |
| 64 | 18 | 16 | 5 | 1 | 29 |  |
| 88 | 14 | 5 | 5 | 0 | 20 |  |
| 62 | 13 | 4 | 5 | 0 | 19 |  |
| 37 | 12 | 12 | 4 | 5 | 17 |  |
| 58 | 6 | 2 | 5 | 0 | 4 |  |
| 75 | 13 | 5 | 5 | 0 | 10 |  |
| 87 | 21 | 22 | 5 | 4 | 3 |  |
| 75 | 30 | 30 | 5 | 3 | 2 |  |
| 86 | 18 | 26 | 4 | 0 | 10 |  |
| 69 | 22 | 6 | 3 | 1 | 8 |  |
| 66 | 6 | 27 | 5 | 0 | 15 |  |
| 52 | 30 | 5 | 5 | 1 | 12 |  |
| 78 | 13 | 14 | 4 | 1 | 9 |  |
| 77 | 15 | 24 | 5 | 2 | 3 |  |
| 59 | 24 | 30 | 5 | 2 | 18 |  |
| 59 | 21 | 28 | 5 | 1 | 11 |  |
| 76 | 30 | 30 | 3 | 6 | 5 |  |
| 82 | 5 | 2 | 3 | 1 | 7 |  |
| 39 | 4 | 2 | 2 | 0 | 7 |  |
| 54 | 1 | 2 | 2 | 0 | 8 |  |
| 75 | 5 | 3 | 4 | 1 | 6 |  |
| 66 | 19 | 27 | 5 | 3 | 30 |  |
| 72 | 9 | 10 | 4 | 37 | 30 |  |
| 69 | 8 | 8 | 4 | 1 | 12 |  |
| 80 | 16 | 22 | 5 | 1 | 8 |  |
| 79 | 21 | 28 | 4 | 3 | 16 |  |
| 38 | 9 | 28 | 4 | 1 | 8 |  |
| 47 | 20 | 6 | 3 | 1 | 9 |  |
| 76 | 13 | 15 | 3 | 1 | 17 |  |
| 58 | 14 | 7 | 5 | 1 | 10 |  |
| 79 | 26 | 16 | 5 | 0 | 23 |  |
| 75 | 9 | 30 | 4 | 1 | 31 |  |
| 74 | 12 | 27 | 4 | 1 | 16 |  |
| 59 | 13 | 12 | 5 | 1 | 13 |  |
| 72 | 17 | 27 | 4 | 7 | 33 |  |
| 40 | 21 | 21 | 4 | 1 | 22 |  |
| 72 | 14 | 32 | 5 | 1 | 14 |  |
| 78 | 17 | 25 | 5 | 12 | 56 | 12 |
| 63 | 16 | 17 | 5 | 9 | 19 | 9 |
| 74 | 24 | 25 | 4 | 16 | 32 |  |
| 55 | 18 | 32 | 4 | 2 | 18 |  |
| 55 | 6 | 16 | 4 | 12 | 36 | 17 |
| 50 | 14 | 14 | 3 | 14 | 13 | 14 |
| 39 | 11 | 30 | 3 | 9 | 11 | 9 |
| 58 | 11 | 3 | 5 | 0 | 7 |  |
| 93 | 23 | 30 | 5 | 2 | 1 |  |
| 61 | 21 | 25 | 5 | 8 | 15 | 11 |
| 91 | 17 | 12 | 4 | 0 | 6 |  |
| 94 | 12 | 16 | 4 | 0 | 4 |  |
| 74 | 14 | 17 | 5 | 17 | 33 | 19 |
| 97 | 18 | 25 | 2 | 0 | 3 |  |
| 81 | 4 | 0 | 4 | 0 | 7 |  |
| 79 | 13 | 3 | 3 | 0 | 8 |  |
| 79 | 7 | 2 | 5 | 0 | 6 |  |
| 84 | 23 | 12 | 5 | 0 | 3 |  |
| 65 | 17 | 6 | 5 | 0 | 9 |  |
| 84 | 25 | 25 | 3 | 15 | 14 | 15 |
| 88 | 8 | 5 | 4 | 0 | 6 |  |
| 88 | 11 | 11 | 4 | 1 | 11 |  |
| 77 | 14 | 5 | 5 | 0 | 7 |  |
| 76 | 17 | 15 | 5 | 1 | 9 |  |
| 89 | 16 | 24 | 5 | 1 | 3 |  |
| 69 | 16 | 3 | 4 | 0 | 4 |  |
| 88 | 10 | 16 | 4 | 0 | 9 |  |
| 92 | 11 | 20 | 5 | 7 | 10 |  |
| 91 | 21 | 19 | 4 | 0 | 19 |  |
| 72 | 10 | 22 | 5 | 0 | 2 |  |
| 94 | 20 | 17 | 5 | 1 | 13 |  |
| 84 | 19 | 22 | 5 | 7 | 17 |  |
| 65 | 19 | 6 | 5 | 1 | 19 |  |
| 82 | 17 | 17 | 5 | 0 | 6 |  |
| 87 | 21 | 12 | 3 | 0 | 7 |  |
| 71 | 10 | 4 | 2 | 0 | 4 |  |
| 79 | 4 | 1 | 5 | 0 | 8 |  |
| 79 | 16 | 11 | 4 | 1 | 24 |  |
| 85 | 7 | 19 | 4 | 0 | 8 |  |
| 82 | 12 | 2 | 5 | 0 | 12 |  |
| 80 | 6 | 16 | 5 | 0 | 4 |  |
| 64 | 24 | 8 | 5 | 0 | 26 |  |
| 86 | 24 | 24 | 4 | 2 | 3 |  |
| 77 | 14 | 16 | 5 | 0 | 26 |  |
| 76 | 20 | 14 | 5 | 2 | 9 |  |
| 77 | 21 | 19 | 4 | 0 | 20 |  |
| 75 | 12 | 8 | 4 | 1 | 21 |  |
| 79 | 18 | 18 | 5 | 6 | 8 | 8 |
| 88 | 17 | 14 | 3 | 0 | 16 |  |
| 75 | 16 | 12 | 5 | 0 | 11 |  |
| 84 | 14 | 18 | 3 | 0 | 12 |  |
| 51 | 6 | 4 | 5 | 0 | 24 |  |
| 85 | 9 | 8 | 5 | 0 | 14 |  |
| 48 | 20 | 19 | 5 | 25 | 20 | 25 |
| 79 | 20 | 22 | 5 | 0 | 6 |  |
| 77 | 15 | 2 | 5 | 0 | 7 |  |
| 75 | 24 | 32 | 5 | 2 | 1 |  |
| 92 | 23 | 9 | 5 | 0 | 8 |  |
| 80 | 14 | 15 | 5 | 9 | 27 |  |
| 71 | 14 | 16 | 3 | 1 | 13 |  |
| 75 | 8 | 1 | 5 | 0 | 5 |  |
| 80 | 18 | 21 | 5 | 1 | 22 |  |
| 63 | 19 | 20 | 5 | 0 | 4 |  |
| 63 | 20 | 23 | 3 | 24 | 18 | 24 |
| 82 | 8 | 7 | 5 | 0 | 18 |  |
| 67 | 20 | 4 | 5 | 0 | 7 |  |
| 88 | 20 | 25 | 5 | 1 | 4 |  |
| 81 | 18 | 11 | 5 | 0 | 43 |  |
| 80 | 24 | 16 | 5 | 1 | 12 |  |
| 82 | 17 | 6 | 4 | 0 | 8 |  |
| 62 | 15 | 13 | 5 | 0 | 22 |  |
| 84 | 19 | 20 | 2 | 0 | 18 |  |
| 79 | 3 | 0 | 5 | 0 | 10 |  |
| 72 | 15 | 4 | 5 | 0 | 7 |  |
| 88 | 25 | 12 | 5 | 0 | 5 |  |
| 74 | 27 | 20 | 5 | 1 | 22 |  |
| 80 | 9 | 13 | 5 | 1 | 7 |  |
| 75 | 18 | 12 | 5 | 1 | 22 |  |
| 65 | 34 | 34 | 5 | 5 | 5 |  |
| 80 | 15 | 12 | 5 | 1 | 28 |  |
| 78 | 22 | 20 | 4 | 0 | 24 |  |
| 77 | 18 | 22 | 5 | 0 | 24 |  |
| 84 | 10 | 9 | 4 | 0 | 27 |  |
| 82 | 21 | 21 | 2 | 0 | 7 |  |
| 82 | 3 | 4 | 5 | 0 | 7 |  |
| 45 | 16 | 10 | 4 | 1 | 4 |  |
| 71 | 10 | 14 | 5 | 1 | 21 |  |
| 72 | 18 | 34 | 4 | 3 | 23 |  |
| 80 | 22 | 13 | 4 | 2 | 18 |  |
| 76 | 8 | 4 | 4 | 1 | 11 |  |
| 76 | 9 | 13 | 5 | 8 | 19 |  |
| 70 | 15 | 17 | 3 | 1 | 26 |  |
| 61 | 9 | 3 | 5 | 0 | 7 |  |
| 74 | 20 | 18 | 5 | 1 | 4 |  |
| 87 | 21 | 21 | 5 | 0 | 6 |  |
| 87 | 20 | 17 | 4 | 1 | 18 |  |
| 77 | 10 | 27 | 5 | 1 | 1 |  |
| 84 | 21 | 17 | 5 | 0 | 7 |  |
| 71 | 27 | 27 | 5 | 5 | 5 |  |
| 78 | 21 | 21 | 4 | 1 | 27 |  |
| 83 | 14 | 4 | 5 | 0 | 21 |  |
| 76 | 16 | 25 | 4 | 1 | 1 |  |
| 86 | 8 | 23 | 4 | 3 | 5 |  |
| 76 | 8 | 23 | 4 | 1 | 1 |  |
| 78 | 8 | 7 | 3 | 1 | 13 |  |
| 85 | 9 | 13 | 3 | 0 | 13 |  |
| 76 | 7 | 9 | 5 | 1 | 15 |  |
| 78 | 21 | 19 | 3 | 0 | 6 |  |
| 73 | 7 | 7 | 4 | 1 | 8 |  |
| 72 | 15 | 20 | 4 | 6 | 4 |  |
| 84 | 13 | 15 | 4 | 1 | 5 |  |
| 78 | 20 | 10 | 5 | 2 | 21 |  |
| 30 | 32 | 13 | 5 | 1 | 20 |  |
| 86 | 19 | 24 | 2 | 1 | 13 |  |
| 76 | 3 | 20 | 5 | 6 | 7 |  |
| 81 | 20 | 19 | 5 | 1 | 24 |  |
| 61 | 16 | 27 | 3 | 0 | 3 |  |
| 80 | 5 | 2 | 4 | 0 | 6 |  |
| 62 | 18 | 28 | 5 | 1 | 8 |  |
| 68 | 42 | 6 | 3 | 1 | 7 |  |
| 63 | 9 | 7 | 5 | 1 | 8 |  |
| 65 | 20 | 16 | 5 | 1 | 23 |  |
| 60 | 16 | 11 | 5 | 0 | 6 |  |
| 66 | 16 | 7 | 5 | 0 | 8 |  |
| 74 | 22 | 11 | 5 | 1 | 9 |  |
| 69 | 22 | 27 | 5 | 18 | 29 |  |
| 77 | 17 | 31 | 3 | 1 | 8 |  |
| 61 | 6 | 3 | 4 | 0 | 3 |  |
| 85 | 12 | 21 | 5 | 3 | 1 |  |
| 77 | 17 | 19 | 5 | 7 | 13 |  |
| 88 | 19 | 22 | 5 | 1 | 13 |  |
| 87 | 13 | 2 | 4 | 5 | 15 |  |
| 57 | 14 | 1 | 5 | 0 | 5 |  |
| 79 | 22 | 22 | 5 | 0 | 5 |  |
| 80 | 19 | 24 | 5 | 10 | 18 |  |
| 81 | 17 | 27 | 4 | 1 | 12 |  |
| 80 | 15 | 8 | 5 | 0 | 10 |  |
| 78 | 21 | 19 | 4 | 1 | 7 |  |
| 71 | 21 | 20 | 5 | 0 | 25 |  |
| 73 | 24 | 23 | 5 | 10 | 29 | 10 |
| 74 | 17 | 18 | 4 | 1 | 37 |  |
| 71 | 24 | 30 | 4 | 4 | 9 |  |
| 66 | 24 | 20 | 5 | 9 | 18 |  |
| 64 | 22 | 29 | 5 | 2 | 10 |  |
| 68 | 30 | 30 | 5 | 6 | 14 | 8 |
| 47 | 14 | 30 | 5 | 10 | 14 | 10 |
| 85 | 30 | 30 | 5 | 2 | 12 |  |
| 74 | 30 | 30 | 5 | 10 | 11 |  |
| 74 | 4 | 28 | 5 | 9 | 9 | 9 |
| 83 | 10 | 30 | 5 | 2 | 19 |  |
| 70 | 11 | 19 | 3 | 7 | 28 | 7 |
| 63 | 3 | 6 | 4 | 1 | 6 |  |
| 84 | 4 | 6 | 5 | 0 | 11 |  |
| 68 | 34 | 26 | 3 | 9 | 13 |  |
| 79 | 12 | 29 | 5 | 1 | 5 |  |
| 78 | 9 | 32 | 5 | 3 | 30 |  |
| 46 | 22 | 30 | 5 | 3 | 3 |  |
| 73 | 24 | 30 | 5 | 5 | 19 |  |
| 73 | 15 | 15 | 4 | 1 | 22 |  |
| 73 | 17 | 26 | 5 | 2 | 7 |  |
| 86 | 26 | 27 | 5 | 1 | 10 |  |
| 74 | 25 | 28 | 4 | 1 | 22 |  |
| 69 | 16 | 10 | 4 | 1 | 9 |  |
| 64 | 21 | 29 | 5 | 1 | 25 |  |
| 76 | 16 | 30 | 5 | 1 | 14 |  |
| 88 | 7 | 32 | 5 | 2 | 15 |  |
| 76 | 17 | 25 |  | 3 | 9 |  |
| NIHSS: National Institute of Health Stroke Scale; mRS: modified Rankin scale | | | | | | |
